# Green Approaches for Sustainable Development of Liquid Separation Membrane

**DOI:** 10.3390/membranes11040235

**Published:** 2021-03-25

**Authors:** Wei Jie Lee, Pei Sean Goh, Woei Jye Lau, Ahmad Fauzi Ismail, Nidal Hilal

**Affiliations:** 1 Advanced Membrane Technology Research Centre, School of Chemical & Energy Engineering, Faculty of Engineering, Universiti Teknologi Malaysia, Skudai 81310, Johore, Malaysia; weijie934@hotmail.com (W.J.L.); wjlau@petroleum.utm.my (W.J.L.); afauzi@utm.my (A.F.I.); 2 Water Research Centre, New York University Abu Dhabi (NYUAD), Saadiyat Marina District, Abu Dhabi PO Box 129188, United Arab Emirates

**Keywords:** sustainable membrane desalination, environmental friendliness, green solvents, solvent free, click chemistry, chemical vapor deposition, plasma treatment

## Abstract

Water constitutes one of the basic necessities of life. Around 71% of the Earth is covered by water, however, not all of it is readily available as fresh water for daily consumption. Fresh water scarcity is a chronic issue which poses a threat to all living things on Earth. Seawater, as a natural resource abundantly available all around the world, is a potential water source to fulfil the increasing water demand. Climate-independent seawater desalination has been touted as a crucial alternative to provide fresh water. While the membrane-based desalination process continues to dominate the global desalination market, the currently employed membrane fabrication materials and processes inevitably bring adverse impacts to the environment. This review aims to elucidate and provide a comprehensive outlook of the recent efforts based on greener approaches used for desalination membrane fabrication, which paves the way towards achieving sustainable and eco-friendly processes. Membrane fabrication using green chemistry effectively minimizes the generation of hazardous compounds during membrane preparation. The future trends and recommendations which could potentially be beneficial for researchers in this field are also highlighted.

## 1. Introduction

As the demand for fresh water surges due to population and economic growth, the development of agricultural activities, industrialization and a decrease in available fresh water supply, desalination has become a promising technology to produce fresh water [1]. Desalination converts water with a high salt concentration, such as saline water, brine water, and brackish water, into fresh water. Over the last decade, desalination has become increasingly important around the world with significant growth in Asia and the Americas [2]. The majority of the installed desalination plants are located in the Middle East where seawater is plentiful and fresh water is scarce [3]. Desalination technologies can mainly be classified into thermal-based and membrane-based desalination [4,5,6]. While there are multiple ways to operate these desalination technologies, the systems being commercially employed are usually multi-stage flash (MSF), reverse osmosis (RO), and multi-effect distillation (MED) due to their high desalination efficiency [7,8]. Nonetheless, the preparation materials used and brine waste produced throughout the processes could impose adverse environmental pollution, making the whole process unsustainable and not environmentally friendly.

Membrane-based desalination processes are gaining worldwide attention due to their unparalleled advantage over their traditional counterparts. Membrane-based desalination are accounted for 60% of total desalination plants [9,10]. Compared to the traditional desalination technologies, membrane technologies are well recognized for its high selectivity, low carbon footprint, reduced energy expenditure, and minimized usage of auxiliary chemicals to carry out the process [11]. Among the membrane desalination technologies, RO and nanofiltration (NF) are classified as pressure-driven processes whereby hydraulic pressure is applied to overcome the osmotic pressure and thus effectively remove solutes from a highly concentrated feed solution (FS) [12]. These pressure-driven processes are also energy-intensive, whereby its power consumption could range from 2 to 5 kW·h·m^−3^ depending on the nature of FS [13,14]. Membrane distillation (MD) involves the vaporization of water at a temperature lower than the boiling point, whereby vapor is passed through a hydrophobic membrane retaining the liquid phase solutes at the feed side. The heating process is energy intensive and requires much more than that of RO and NF [15,16]. As the energy consumption which accounts for a significant portion of the total operating cost is the major bottleneck of desalination processes, various efforts are underway to address this limitation. For instance, installing an efficient pre-treatment system and energy recovery devices has remarkably reduced the energy expenses of the desalination process [17,18,19]. Various alternative desalination processes have also been explored for the same purpose. Forward osmosis (FO), which utilizes an osmotic pressure gradient as a driving force to separate pure water from FS using a more concentrated draw solution (DS), stands out as an emerging process for desalination. While a single unit of FO is impractical to obtain desalinated drinking water, FO has been feasibly coupled with RO, NF and/or MD as the pre-treatment process aiming to desalinate water at a lower energy cost [20,21,22].

The preparation of a polymeric membrane for desalination usually requires two steps, which are substrate preparation followed by the active layer coating atop the substrate. Either way, coating can sometimes be unnecessary, depending on the type of application. In the substrate preparation stage, several methods such as phase inversion, electrospinning, stretching and sintering are employed [23]. Phase inversion and electrospinning require solvents while the rest do not. This review focused on phase inversion or phase separation, as the most common and principal method for substrate preparation. During phase inversion, a substrate in the pore size range of ultrafiltration (UF) is produced [24,25,26]. To prepare a membrane with a finer pore size range for the application of NF, RO, and FO, a thin film composite (TFC) membrane is made with an active layer coated substrate. Meanwhile, the MD application fundamentally requires an asymmetric membrane which is usually modified with hydrophobic coating. To fabricate the TFC membrane, the active layer coating can be done in several means, with interfacial polymerization (IP) as the most common way. Meanwhile, for MD hydrophobic coating, there are methods such as low surface tension coating, and/or the employment of nanostructures to increase surface roughness [27].

Although membrane technologies in general have been acknowledged as eco-friendly technologies compared to other contemporary separation technologies, it is not always the case in membrane preparation. During the membrane preparation process, large amounts of toxic solvents such as *N*-methyl-2-pyrrolidone (NMP), *N*,*N*-dimethylacetamide (DMA), *N*,*N*-dimethylformamide (DMF) and tetrahydrofuran (THF) are often used [28]. These solvents pose potential risks related to hazardousness and toxicity towards health and the environment. The fabrication of polymeric membrane induces an environmental issue which limits operation in the long run. In light of increasingly severe environmental issues, the recent development of membrane desalination has been progressively transformed to achieve better sustainability. From the environmental point of view, a process is deemed sustainable if it does not cause severe pollution which might threaten lives. The preferred solution in achieving sustainability follows the sequence of reduction, reuse, recycling, treatment, and disposal. The former three options, reduce, reuse and recycle, or commonly known as the 3Rs, uphold the ideology of circular economy [29]. In most cases, reuse is a more favorable option compared to recycling as recycled resources would still go through another production stage whereby energy and materials are further expended. Ultimately, reducing is the most preferable approach as the reduced resources usage can save in terms of the efforts needed for all the other steps [30].

A thorny dilemma is that throughout the entire membrane preparation process, the use of solvents which are toxic to the environment is inevitable. A green membrane fabrication process promotes sustainability and prolongs the process. Applying green chemistry to membrane fabrication serves as a good way of reducing the use of resources, which complies with the concept of 3Rs. It potentially minimizes the amount of hazardous waste created to be disposed, recycled and reused. In this review, the application of green solvents and solvent-free methods for membrane preparation as some green alternatives for the assurance of environmental friendliness are discussed. Green solvents are those characterized by non-toxicity, low volatility, and/or derivatives of renewable sources. Complying with the Twelve Principles of Green Chemistry which state that conventional solvents should be replaced with low-toxicity, recyclable, inert, abundant, easily separable, green solvents, or be solventless, could then pave the way to pollution-free desalination as the world is facing water shortage challenges and naturally occurring resources like seawater are the best place to channel water sources [31].

Kim et al. [32] reviewed the recent progress in improving the sustainability of membrane fabrication using green solvents and chemicals. The recently identified green solvents with their novel procedures of employment for the fabrication of a high-performance membrane have been documented. Figoli et al. [24] reviewed the application of a wide range of solvents in membrane fabrication. Twelve green principles were provided to identify the toxicity level of solvents, and also to identify whether a novel solvent is deemed a green solvent that does not adversely impact the environment.

Despite all the previous efforts, a comprehensive review covering the complete membrane preparation process, from substrate fabrication to membrane modification and post-treatment using a greener approach, is still lacking. This paper provides a comprehensive review which involves all of them, granting a better understanding and insight to readers as a whole. In addition, the literature summarized in their studies mainly comprises dye and protein rejections as applications below the NF range. In this review, the scope is focused on membrane production for application related to desalination application (NF to FO range). This review serves as a foundation for future studies, giving readers valuable literature and a more updated knowledge for understanding the current trend in sustainable ways of membrane preparation, in turn inspiring some new ideas and creating new insights for more innovations. In this contribution, the discussion was conducted in a progressive manner, whereby the substrate preparation stage using green solvents was discussed first, followed by solvent-free methods for membrane modification. Finally, the review concludes with future challenges on several prospects and concise conclusions.

## 2. Substrate Preparation via Phase Inversion

Ideally, substrate preparation without solvents would be the most perfect way to promote environmental cleanliness. Nonetheless, for decades, the majority of membranes are prepared through solution-based process, mainly phase inversion. Switching the solution-based process to a non-solvent method would not enable maintaining the surface area production on par, at least for the current state. There is, however, a method such as 3D printing for micro and nanofabrications of UF and ceramic membranes, with the limitation of poor resolution which is to date unable to be scaled up to be commercially practical [33,34]. Three components are typically involved during phase inversion, namely the solvent, non-solvent, and polymers. The process, in brief, follows the sequence of the dissolution of polymers in the solvent, followed by casting on a support, and then immersion in a non-solvent coagulation bath. Phase inversion occurs as a result of the phase change of the dope solution or casting solution, comprising a homogeneously dissolved polymer in a solvent, which gives rise to polymer-poor and polymer-rich phases during the solidification process. Polymer-poor phases will form pores while polymer-rich phases will become the support frame of the membrane [35]. The phase separation relies on the presence of a miscibility gap in the phase diagram [36]. The solution is unstable in the miscibility gap, hence liquid–liquid separation easily occurs forming a membrane structure (derived from polymer-rich phase) with pores (derived from the polymer-poor phase). Phase inversion can be generally classified into several types, such as temperature-induced phase separation (TIPS), non-solvent induced phase separation (NIPS), evaporation-induced phase inversion (EIPS), and vapor-induced phase inversion (VIPS) [37]. Among them, TIPS and NIPS are among the most popular substrate fabrication techniques for membrane-based desalination, which will be the focus the of this study [24].

**Figure 1:**
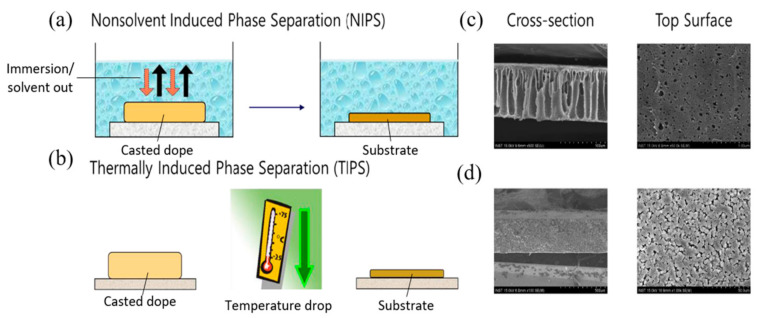
Phase inversion using: (**a**) NIPS; (**b**) TIPS with their respective (**c**); (**d**) cross-section and top surface morphologies [32].

In TIPS, the polymer is dissolved in a solvent commonly known as a latent solvent which only works at a high temperature close to the melting point of a polymer, forming a homogeneous solution [38,39]. The homogeneous dope solution is then immersed in a water bath and the decrease in temperature yields polymer precipitation. Depending on the temperature required, the whole process can be more energy intensive than NIPS. However, it is very effective in fabricating polymeric membranes with various morphologies [36,40]. By manipulating the temperature, the pore size of the membrane can be controlled significantly and hence the membrane with less defect formation, macrovoid absence, high mechanical strength, as well as isotropic and anisotropic structures, are possible to be prepared [41,42,43,44]. In NIPS, the polymer is allowed to dissolve in the solvent via stirring until a homogeneous dope solution is formed. It is then casted on a glass plate (flat sheet) or extruded through a spinneret (hollow fiber) followed by immersion in a non-solvent water bath. The exchange of a solvent allows the polymer precipitation forming membrane. The solvents used in NIPS can generally dissolve the polymer at room temperature [45,46]. Macrovoids and finger-like structures are formed within the membrane due to solvent/non-solvent exchange, resulting in weak mechanical strength. The membrane can rupture easily when working in a long-term process under high temperature. Figure 1 schematically illustrates the membrane fabrication through NIPS and TIPS with their respective membrane morphologies.

### 2.1. Green Solvents as Alternatives forTraditional Solvents

It is worth noting that a lot of the commercial organic solvents used in polymer dissolution are toxic and harmful to the environment. For instance, the solvents traditionally employed for NIPS include NMP, DMA, DMF, THF, and 1,4-dioxane [47], while for TIPS these are methyl salicylate [48], phthalates [49,50], and diphenyl ether [51,52]. Table 1 shows the hazard classification of the toxic solvents commonly used for membrane preparation. Upon disposal into environment, these pose a dangerous risk to humans. At the industrial level, 95% of the total waste generated during membrane fabrication is accounted for by the wastewater contamination of these solvents [53]. It is eloquently stated that the amount of solvent used in a process can affect the magnitude of the health impact on humans [54]. Hence, lots of companies have taken the recycling of wastewater containing solvent into consideration by adopting methods such as adsorption or membrane separation [55]. Nonetheless, whilst solvent recycling is highly desirable, it is not always possible due to the quality requirement such as high purity and regulatory demand [56,57]. Many very large scale processes (bulk chemical synthesis) which involve solvents require “disposal solvents” that can be incinerated after being used, such as toluene.

The growing awareness of the adverse impacts of toxic solvents has encouraged the use of non-toxic solvents as green alternatives to improve the sustainability of membrane preparation. An ongoing challenge emerges while replacing traditional solvents with green solvents. Water would make the greenest choice of solvent when it comes to availability and non-toxicity. Though it could be an interesting concept with huge potential, its practicability is rather restricted. Kamp et al. [58] utilized pure water as the solvent to induce phase separation and found out that phase separation and membrane formation depend solely on a change in pH as the driving force. Moreover, the process itself is unstable, and the membrane did not yield satisfactory performance. The green solvents selected in this study are not merely involved in the dissolution of the polymers, but must also serve the purpose of creating a membrane with desired performance and morphologies. During the membrane formation process, it is important to take note that solvent characteristics such as boiling point, dielectric constant, polarity, and viscosity could significantly affect the membrane characteristics.

**Figure 2:**
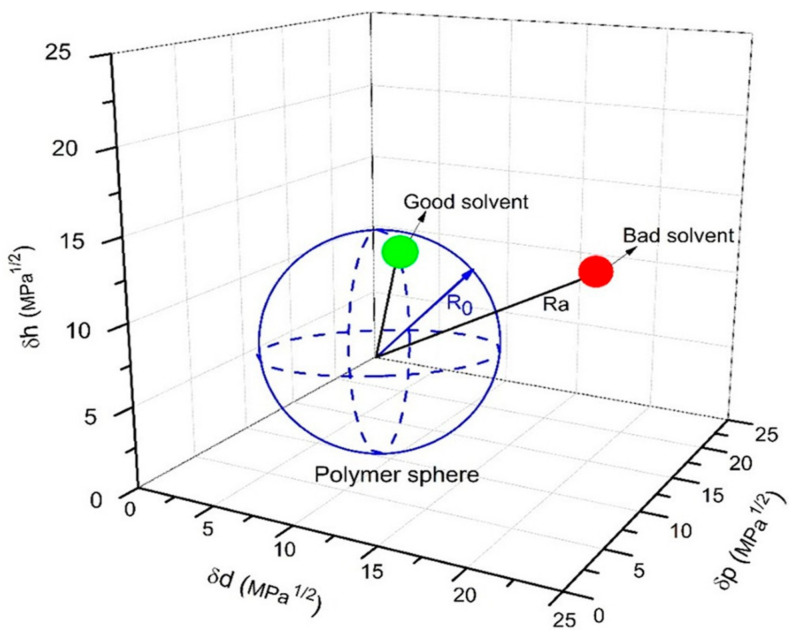
Interaction radius of the Hansen solubility sphere (R_0_) for the radius determination of the good and bad solvents [60].

**Figure 3:**
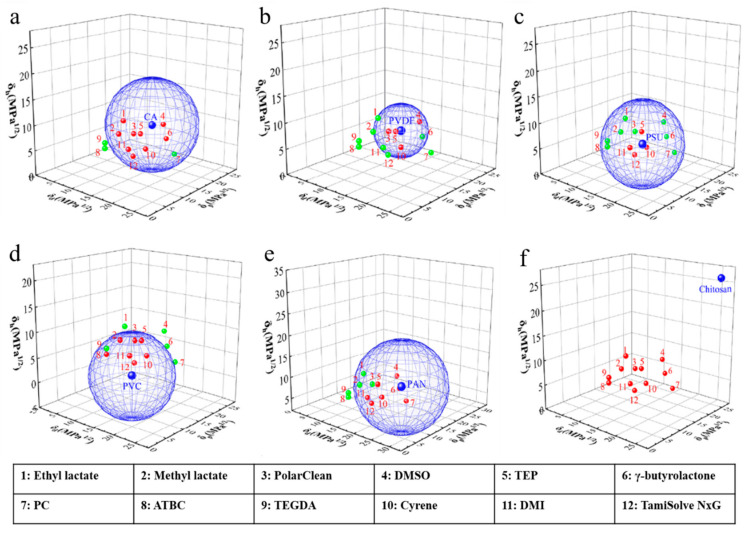
3D Hansen solubility parameter (HSP) spheres of green solvents and typical polymers (red dots indicate the inside sphere, green dots indicate the outside sphere) [61].

Green solvents which are either chemically produced or naturally occurring have been discovered to be the promising candidates. For NIPS, dimethyl sulfoxide (DMSO), methyl lactate, ethyl lactate, and ionic liquids are some good non-toxic solvents to replace traditional ones; while for TIPS, acetyltributylcitrate (ATBC), acetyltriethylcitrate (ATEC), triacetin and soybean oil are some good alternatives. In this study, all the green solvents were categorized into synthetic organic solvents, bio-sourced solvents, and ionic liquids regardless of their fabrication technique. As solvents have different physicochemical properties that tend to interact with the polymer and the fillers leading to various membrane morphologies and properties, it is crucial to determine whether solvent replacement is able to maintain or achieve a better membrane performance instead of merely following the sustainable development agenda. Generally, a green solvent must be in compliance with a few principles of green chemistry, which are: (i) Safer solvents and auxiliaries: The use of auxiliary substances (e.g., solvents, separation agents) should be made unnecessary wherever possible and innocuous when used; (ii) Use of renewable feedstocks: A raw material or feedstock should be renewable rather than depleting whenever technically and economically practicable; (iii) Inherently safer chemistry for accident prevention: Substances and the form of a substance used in a chemical process should be chosen to minimize the potential for chemical accidents, including releases, explosions, and fires [59]. As most of the solvents used in membrane preparation are water insoluble, it is important to prepare an organic extraction bath to remove the excess solvents from the membrane. Despite the manifold advantages, in membrane desalination, the use of these green solvents is still scarce. Instead, they usually lead to preparing membranes for protein, sugar, or dye removal at the current state:

To execute solvent-based phase inversion perfectly, the prerequisite would be the complete dissolution of polymer. The affinity between polymer and solvent is hence an important factor that determines the phase separation process and the resulting membrane performance. It is critical to choose green solvent with good affinity towards the polymer to ensure proper dissolution. Ra value which is calculated by the Hansen solubility parameter (HSP) using the equation below can be used to describe such affinity:

(1) $$R_{a}=\sqrt{4{\left(\delta_{1}-\delta_{d2}\right)}^{2}+{\left(\delta_{p1}-\delta_{p2}\right)}^{2}+{\left(\delta_{h1}-\delta_{h2}\right)}^{2}}$$

whereby δ_d_ is the dispersion parameter, δ_p_ is the polar parameter, and δ_h_ is the hydrogen bonding parameter. The lower the R_a_ value, the better the polymer−solvent compatibility, and the polymer will most probably be soluble in that solvent, as depicted in Figure 2. R_0_ value can be used to indicate the Hansen solubility sphere of a polymer. In order to have good affinity towards a polymer, the R_a_ value of a solvent must be smaller than R_0_. Table 2 and Figure 3 further summarize the HSP values of some green solvents and typical polymers used in substrate fabrication. Red dots represent that R_a_ < R_0_ (inside sphere), while green dots represent that R_0_ < R_a_ (outside sphere). 

### 2.2. Synthetic Organic Solvents

#### 2.2.1. DMSO

Highly volatile organic compound (VOC) solvents could emit toxic vapor harmful to the environment. DMSO as a low VOC solvent owing to its high boiling point (189 °C at 1 atm) and low vapor pressure (0.6 mmHg at 25 °C) has low toxicity. It also has a very similar polarity and solvency power to NMP, DMF, and DMAC, which can effectively dissolve many types of polymers such as polysulfone (PSU) and polyvinylidene fluoride (PVDF), making it a good candidate for toxic solvent replacement [70]. In some studies, DMSO was reported to be a green, highly polar and water miscible organic solvent which does not have a harmful impact on the environment [24,72]. DMSO is made of lignin, a natural compound from tree cells which acts as glue giving to their mechanical structure. DMSO has the unique feature of being able to be quickly absorbed by human tissues, hence possessing the risk of carrying toxin on the skin. Nonetheless, if the solvent choice is mainly made in function of peculiar toxicity for human health, DMSO appears as a good replacement for more toxic solvents according to the principle of green chemistry stating “Safer solvents and auxiliaries: The use of auxiliary substances (e.g., solvents, separation agents, etc.) should be made unnecessary wherever possible and innocuous when used” [24]. The only shortcoming is its unpleasant odor, however, it has been significantly improved in the new version of DMSO EVOL^TM^ [73].

**Figure 4:**
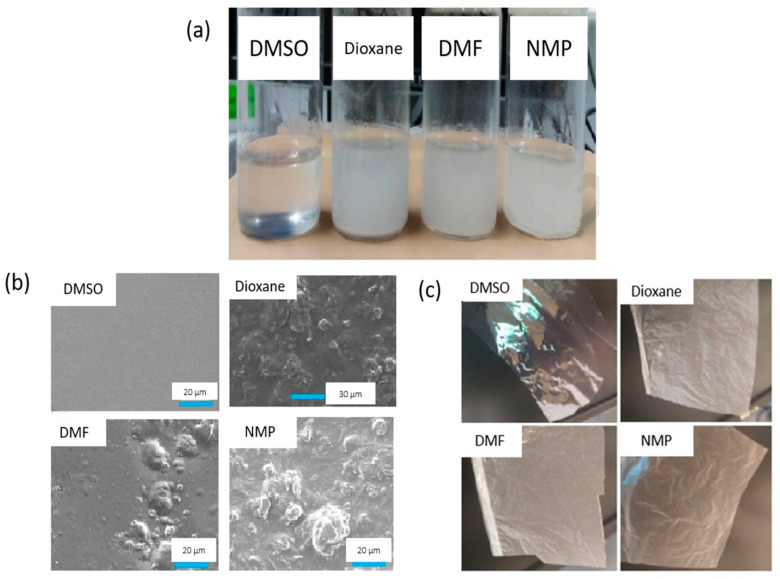
Labelled diagrams of: (**a**) Cellulose nanocrystals (CNCs) suspension in different solvents; (**b**) SEM images of nanocomposite membranes formed from different solvents; and (**c**) photographs of nanocomposite membranes formed from different solvents [74].

Apart from lower toxicity, it is also important to determine to what extent solvent change can affect the performance of the membrane. Prihatiningtyas et al. [74] compared the membrane desalination performance of DMSO to several traditional solvents which are NMP, DMF and dioxane. Cellulose nanocrystals (CNCs)-incorporated cellulose triacetate (CTA) membranes were successfully fabricated via dope casting in different solvents. Among all the solvents, the DMSO-based membrane successfully outshined with a homogeneous distribution of CNCs on the membrane surface and decent performance. CNCs are a polar nanomaterial with surface charges and electrostatic repulsion interactions between the CNCs rods. Hence, compared to the other solvents, the strong polarity of DMSO (δp = 16.4 MPa1/2) potentially donates a strong ionic strength in the suspension, of which the resulting repulsion hinders CNC aggregation and is attributed to the homogeneous dispersion of CNCs. Consistent results can be observed from Figure 4a, in which the only dope solution with DMSO solvent yields a clear solution, indicating the homogeneous distribution of CNCs, while the rest result in a rather cloudy solution. The resulting membranes (Figure 4c) prepared from four solvents also showed a similar outcome, with DMSO-based nanocomposite membrane having a transparent appearance while the other showing an opaque display. Meanwhile, Figure 4b indicates that the surface of the DMSO-based nanocomposite membrane was the smoothest among all membranes due to the homogeneous distribution of CNCs, making water diffusion much easier with lower fouling propensity. The membrane provided decent water flux (11 kg·m^−2^·h^−1^) without sacrificing the NaCl rejection (99.9%) using 30 g·L^−1^ of NaCl solution as FS. Meanwhile, for a high FS concentration (90 g·L^−1^ of NaCl), the NMP-based and dioxane-based membranes suffered from salt rejection and membrane leakage.

After that, the same group of researchers [75] incorporated alumina (Al_2_O_3_) into CTA membranes also using DMSO as the solvent. It was then determined that alumina has excellent dispersibility in DMSO regardless of its concentration, with 2 wt.% alumina as the optimum concentration significantly improving the membrane hydrophilicity, thermal stability and mechanical strength. The resulting membrane outperformed the pristine CTA membrane with a promising flux enhancement of 204% using 30 g·L^−1^ as FS. Meanwhile, a decent salt rejection of more than 99.8% was obtained. Li et al. [76] fabricated polyamide (PA)/Kevlar aramid nanofiber (ANF) composite membranes for NF desalination. It is worth noting that ANF is a solvent-resistant Kevlar nanofiber. DMSO was again a great solvent able to homogeneously dissolve ANF forming ANF hydrogel dope, and then further fabricated into membrane via NIPS. The facile solvent treatment successfully shifted the desalination performance of the ANF-TFC membrane from RO into NF, which is one order of magnitude higher than the pristine TFC membrane. In terms of membrane characterization and performance, the ANF-TFC membrane exhibited lower surface roughness, lower surface zeta potential and higher surface hydrophilicity. Meanwhile, the salt–water separation performance was outstanding as well, with high rejections for monovalent salt (NaCl, 80.3%) and multivalent salts (MgSO_4_, 99.4%; Na_2_SO_4_, 100%; MgCl_2_, 92.7%). More interestingly, the performance WAs on par with the reference commercial NF membranes (NF270 and NF90) tested in cross-flow filtration.

Wang et al. [77] investigated the effects of different solvents such as DMSO, TEP, DMA, DMF, and mixtures of them on the physicochemical properties of the fabricated membranes. The obtained results indicated that the skin layer formation on the surface of membrane was mainly affected by the equilibrium thermodynamics, while the solvent/non-solvent exchange rate affected the support layer formation. It was reported that using DMSO which has a high polarity value as the solvent, the surface zeta potential of the membrane was more negative than the membranes fabricated using other solvents. The membrane fabricated using a mixture of TEP-DMSO solvent yielded the highest water permeability, followed by a membrane prepared using pure DMSO, accounting for the improved membrane surface negativity. Moreover, the membranes fabricated using DMSO as solvent had a higher porosity with more macrovoids and cellular pores in their structure. Adam et al. [78] and Sener et al. [79] both reported that membrane preparation using DMSO as solvents yielded a thinner active surface layer in comparison to other solvents like DMF and NMP. Hence, in terms of water selectivity and permeability, better performance was obtained. In a nutshell, DMSO has proven itself a promising alternative to replace the traditional toxic solvents for greener membrane fabrication.

#### 2.2.2. Dimethyl Carbonate

Dimethyl carbonate (DMC) was another green solvent used by Shi et al. [80] as a co-solvent joint to tannic acid to fabricate the PSU-TFC membrane for RO desalination. DMC is environmentally benign as the building blocks mainly comprise of CO_2_, which does not emit VOCs to the atmosphere. It was observed that the addition of DMC co-solvent pronouncedly improved the leaf-like structure of the membrane surface which is favorable for solute rejection. The resulting membrane showed outstanding performance obtaining a water flux of 64.2 L·m^−2^·h^−1^ and salt rejection of 99.03%.

#### 2.2.3. TamiSolve NxG

Marino et al. [68] first attempted to use TamiSolve NxG as a green solvent for poly(vinylidene fluoride-hexafluoropropylene) (PVDF-HFP) membrane preparation. Unlike NMP or DMA, TamiSolve NxG is not classified as a developmental-reprotoxic, which does not cause infertility and developmental toxicity in the offspring. Meanwhile, it has high chemical and thermal stability, as well as solvency similar to NMP and DMA towards a wide range of compounds making it a superb choice as a green solvent. The strong solvency of TamiSolve NxG can be further observed from the membrane characterization results, whereby thin membrane (0.109 mm) with small average pore size (~0.04 μm) was formed. Compared to the commercial polypropylene (PP) membrane, the resulting membrane exhibited a similar pore size. More importantly, when it was tested under direct contact membrane distillation (DCMD) desalination, satisfying water flux and rejection performance were obtained. The water flux increased gradually along the temperature, hitting a maximum flux of ~25 L·m^−2^·h^−1^ at 60 °C. A consistent salt rejection of 99.5% was also obtained for three consecutive tests. Table 3 summarizes some previous works from several authors using synthetic organic solvents in membrane fabrication.

### 2.3. Bio-Sourced Solvents

#### 2.3.1. Methyl Lactate

Methyl lactate is a naturally derived ester of lactic acid, also known as lactic acid methyl ether [81]. As the simplest form chiral ether, it is a colorless liquid which shows excellent chemical properties as a non-toxic solvent for CA membrane preparation because it is biodegradable, versatile, and water miscible [69]. It can completely dissolve chloride salts and CA pellets forming a homogeneous solution to be casted. Particularly, methyl lactate is only used in the fabrication of CA UF membrane [24]. The molecular weight cut off (MWCO) of the membrane prepared using methyl lactate is around 10−500 kDa, which represents a typical UF membrane, while the percentage of solutes rejection is similar to the membranes fabricated with NMP as solvent [82,83].

Medina-Gonzalez et al. [59] prepared methyl lactate as the green solvent which is in compliance with a few principles of green chemistry mentioned in the previous section. They evidenced the feasibility of fabricating a CA UF membrane using methyl lactate as a solvent and CaCl_2_ as porogens. With the aid of CaCl_2_ as porogen, the resultant membrane was pressure resistant (up to 5 bar) and defect free, with an MWCO lower than 500 kDa, making it completely possible for the rejection of some bulky-sized salts. Meanwhile, Rasool et al. [69] prepared a CA membrane using methyl lactate as solvent and 2-methyltetrahydrofuran as a co-solvent via NIPS to test for protein and salt rejections. Prior to the casted dope solution immersion in the water bath, some membranes were given extra evaporation period of 30 s. The membranes with 30 s evaporation period had a higher MgSO_4_ rejection but lower water permeation, which was attributed by the membrane densification due to delayed mixing and a slower exchange pace between the solvent and non-solvent. The membrane with respect to best water permeation showed 80.2% of the MgSO_4_ rejection at 12.8 L·m^−2^·h^−1^·bar^−1^, while the membrane with respect to best salt rejection showed 96.5% of MgSO_4_ rejection at 1.1 L·m^−2^·h^−1^·bar^−1^. The water flux can be easily tweaked by increasing the operating pressure within the threshold limit. Judging from the green metrics, the replacement of the conventional solvents by methyl lactate is definitely feasible alongside some benefits brought to the whole process such as reduced eco-toxicity.

#### 2.3.2. γ-Butyrolactone

γ-Butyrolactone, a water-miscible, hygroscopic colorless liquid with a weak odor, is an excellent choice of non-toxic solvent for membrane fabrication. It is the simplest four-carbon lactone. Generally, it is employed as an intermediate in the production of other chemicals, such as NMP. At the same time, it can also be a good replacement of toxic solvent for membrane preparation. Song et al. [84] prepared a PVDF hollow fiber membrane via TIPS using γ-butyrolactone and dioctyl phthalate (DOP) as binary non-toxic solvents for MD. The objective of this study was to predict the possible composition to produce a bi-continuous structure in the microporous membrane. Hence, the solubility parameter theory and Flory–Huggins equation were used to deduce the quantitative relation equations between the phase separation temperature and interaction parameter. Finally, a predicted system comprised of 12.74 wt.% PVDF, 58.44 wt.% DOP and the rest γ-butyrolactone, successfully fabricated hollow fiber membranes with a desired interconnected sponge-like structure. Using 3.5 wt.% of NaCl solution as FS, the membrane performances were studied in DCMD test. The resulting membrane proved superior tp pure water flux (51.5 L·m^−2^·h^−1^), while the salt rejection was able to maintain at 99.99% at all time, indicating the feasibility of the binary solvents’ combination to prepare a hollow fiber MD membrane for desalination application. In addition, Bey et al. [85] reported that γ-Butyrolactone is a good replacement of toxic solvents such as DMA, DMF, and chloroform. Polyether ether ketone (PEEK-WC) hollow fiber membrane was fabricated for a hollow fiber membrane contractor (HFMC). The obtained results exhibited excellent chromium salt rejection.

#### 2.3.3. ATBC

ATBC is made by the esterification of tributylcitrate and acetic acid. In TIPS, it is known as one of the most popular green solvents with low toxicity and high compatibility with several polymers that is being used to replace highly toxic phthalates for membrane preparation. ATBC is a nearly colorless and odorless oily diluent which is mainly used as the plasticizer for polyvinyl chloride, ethyl-cellulose, methacrylic, acrylic, vinyl-acetate, nitrocellulose and urethane polymer systems [86,87]. It is widely employed in the pharmaceutical, medical, food, and toys production industries [88].

Particularly, it can be used to prepare PVDF membrane via TIPS. Cui et al. [88] pioneered the use of ATBC as a green solvent in PVDF membrane preparation. The membranes were prepared in both hollow fiber and flat sheet forms. ATBC was proved to be efficacious in dissolving PVDF from a high concentration to low concentration. The morphological results showed that a high PVDF concentration could potentially improve the membrane mechanical strength but sacrifice the pure water flux. Instead, lowering the PVDF concentration and quenching temperature could enhance the pure water flux. These results potentially open a new avenue in the preparation of PVDF membrane using ATBC as an efficient yet non-toxic solvent. Hassankiadeh et al. [89] then fabricated PVDF hollow fiber membranes with different molecular weight using ATBC as a green solvent in the desalination application. Similarly, the study revealed that quenching temperature significantly affected the membrane performance while the washing temperature had a negligible effect on that. From the DCMD test, it was disclosed that the lower PVDF molecular weight granted high pure water flux due to higher mean pore size and overall porosity. More interestingly, the resulting membranes had even higher water flux in comparison with few types of commercial PVDF membranes. Meanwhile, the membrane prepared with the highest molecular weight despite having a slightly lower water flux, but was able to maintain a high salt rejection of 99.9% using 3.5 wt.% NaCl FS under all operating conditions, indicating the formation of uniform membrane structure using ATBC as a solvent.

**Figure 5:**
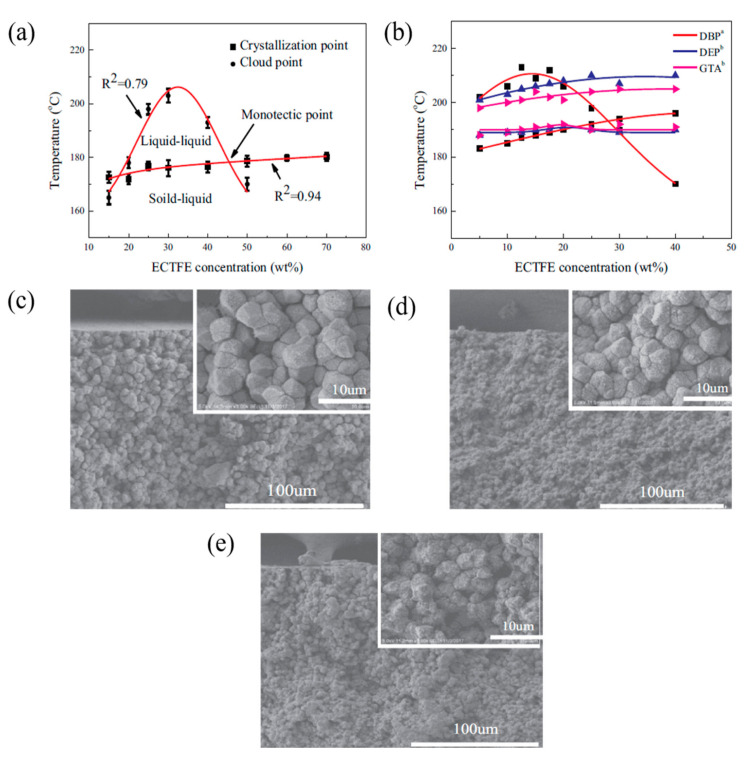
(**a**) Phase diagram of poly(ethylene chlorotrifluoroethylene) (ECTFE)/ATBC; (**b**) phase diagram of ECTFE/glycerol triacetate (GTA), ECTFE/DBP and ECTFE/ diethyl phthalate (DEP); (**c**,**d**) cross-sectional morphologies of ECTFE membrane with a polymer concentration of 20 and 40 wt.% at a quenching temperature of 20 °C; (**e**) cross-sectional morphologies of ECTFE membrane with a polymer concentration of 30 wt.% at a quenching temperature of 80 °C [90].

On the other hand, Xu et al. [90] used ATBC as the solvent to prepare poly(ethylene chlorotrifluoroethylene) (ECTFE) membranes via TIPS for membrane distillation (MD) desalination. Figure 5a shows the binary phase diagram of ECTFE/ATBC, whereby a wide liquid–liquid separation region is observed between ECTFE concentration of around 17 wt.% to 48 wt.%, while solid–liquid phase separation happens outside this ECTFE range. Liquid-liquid separation is more favorable than solid-liquid separation as the former can form a bi-continuous structure which is ideal for water transport, while the latter yields spherulite structure. It was then compared with the phase diagram (Figure 5b) of ECTFE/glycerol triacetate (GTA), ECTFE/DOP and ECTFE/diethyl phthalate (DEP) from other studies in the literature [49,91], before it was noticed that the liquid-liquid phase separation of ECTFE/ATBC was much wider than the other combinations. This indicates a higher compatibility of ECTFE/ATBC compared to the other, attributed by high affinity between the solvent and the polymer. High affinity resulted in a low nucleation rate but higher nuclei growth rate as the nuclei has sufficient space for growth. Furthermore, Figure 5c–d illustrate the cross-sectional morphologies of ECTFE membranes prepared with 20 and 40 wt.% of ECTFE at 20 °C quenching temperature. It can be noticed that a higher polymer concentration would increase the spherulite density, which eventually resulted in a narrower gap between the spherulites, leading to smaller membrane pore size and porosity. However, the ideal bi-continuous structure was not observed owing to the fact that the quenching temperature of 20 °C was too low leading to a high cooling rate, and the separation duration was too short to form a bi-continuous structure. The quenching temperature was increased to 80 °C to prolong the cooling rate. As illustrated in Figure 5e, larger spherulite structure with a tighter gap was produced and eventually intertwined together, forming a bi-continuous structure. In addition, by increasing the concentration of ECTFE, a similar trend was observed in the previous PVDF membrane studies, wherein reduced water fluxes and improved mechanical strength were observed due to the increased membrane integrity. Substantially, the membrane with the most optimal ECTFE loading exhibited a decent pure water flux of 22.3 L·m^−2^·h^−1^ and NaCl rejection of 99.9%, making the ECTFE membrane in MD desalination completely viable with ATBC as an environmentally benign diluent.

#### 2.3.4. Soybean Oil

Soybean is one of the most important agricultural commodities in the world, devoting around USD 50 to the world’s total economy. Soybean also accounts for around 57.03% of all oil crops (including oilseed), making it the top global oil crop [92]. Hence, soybean is highly accessible, and soybean oil has also become one of the most important vegetable oils in the world. More than 96% of the soybean oil produced commercially is used in the food production industry such as margarine, salad oil, cooking oil, and shortening. Solvent extraction is the widely used industrial method of extracting soybean oil as the solvent considering the low oil content of soybean [93]. Its chemical properties, zero toxicity nature, and high accessibility has possibly made it an alternative solvent replacement for membrane application [94]. The first attempt of using soybean oil as the solvent in desalination membrane preparation was reported by Tang et al. [95]. The green solvent was used to prepare isotactic polypropylene (IPP) hydrophobic flat sheet membrane via TIPS. Homogeneous doping solution was able to be formed within 25–32 wt.% of IPP, with 70–80% of the resulting IPP membranes’ pore size ranging from 0.02 to 0.2 μm. Soybean oil proved to be a good solvent for the fabrication of the narrow pore size distribution IPP membrane. The pore size distribution could be slightly narrower by increasing the IPP melt index with a quenching temperature of 20–30 °C. Overall, soybean oil as a solvent yields consistent performance over a quenching temperature range with a minor effect on membrane porosity. It had a nearly insignificant effect on the membrane formation. On the other hand, the IPP membrane with 27 wt.% had the most optimum performance, with a pure water flux of 24.81 L·m^−2^·h^−1^ and an NaCl rejection of >99.9%.

Wang et al. [96] employed non-toxic binary solvents, soybean oil and carnauba wax in a novel fabrication approach for PP membranes via TIPS. Carnauba wax can be easily crystallized and recycled by cooling after being extracted by boiling ethanol. Carnauba wax was the first solvent investigated and determined that the addition of carnauba wax to soybean oil could shift the solid-phase separation into liquid-phase separation. The liquid–liquid phase separation region, whereby the separation of molecules occurs through condensation into a denser phase resembling liquid droplets, can be further expanded by continuously increasing the carnauba wax concentration. A wider liquid–liquid separation region could facilitate the membrane fabrication, producing desired membranes with completely interconnected sponge-like pore structure. Soybean oil was secondly added as the latent solvent and the effects of solvents’ composition on the membrane morphologies and performance were investigated. It is crucial to strike a balance between the mechanical strength of the membrane and water permeation, as a higher carnauba wax ratio can significantly reduce the membrane mechanical strength but improve water permeation due to the increased porosity. The resulting PP membrane had an interconnected sponge-like structure formed, which significantly improved membrane elongation at break. At a carnauba wax/soybean oil ratio of 3/7 with 20 wt.% PP concentration, the resulting membrane exhibited an astonishing performance reaching 41.2 L·m^−2^·h^−1^ water flux and 99.95% salt rejection without any salt leakage being observed. Table 4 shows the summary of previous works from several authors using bio-sourced solvents in membrane fabrication for desalination.

### 2.4. Ionic Liquids

Ionic liquids are another great alternative to replace environmentally harmful solvents. Compared to the volatile solvents, ionic liquids with extremely low vapor pressure produced nearly zero VOCs, hence imposing minimum health risks on the operators. Due to their promising applications in many areas such as catalysis [97], polymer synthesis [98], and battery [99], some industries have started to replace traditional solvents with ionic liquids. However, some controversies have arisen, stating that the toxicity of ionic liquids in water remains inconclusive and the sustainability of their production life cycle assessment has not been thoroughly investigated [100]. There is a broad variety of ionic liquids for different types of applications and polymers, and all of them are made up of positive and negative ions. The cation could be pyridinium, imidazolium, quarternary ammonium or quarternaryphosphonium, while the anion could be halogen, trifluoroborate, triflate, or hexafluorophosphate [101]. Ionic liquids possess strong solvency power to dissolve a high concentration of molecules or even biopolymers with limited solubility in traditional solvents.

It is worth noting that some ionic liquids are harmful, and the production route can be quite hard [102]. Nonetheless, their green credential owing to negligible vapor pressure in contrast to the traditional VOCs, as well as excellent versatility, make them a good choice to replace traditional solvents. In membrane application, Xing et al. [103] used ionic liquid 1-ethyl-3-methylimidazolium acetate with low toxicity as an alternative solvent to prepare a polybenzimidazole (PBI) membrane for nanofiltration. 1-ethyl-3-methylimidazolium acetate is highly miscible in water and liquid at room temperature. Compared to other ionic liquids, 1-ethyl-3-methylimidazolium acetate has strong dissolving power even at low viscosity and water content; it is a great hydrogen bond acceptor. As for DMI, 1-ethyl-3-methylimidazolium acetate ([EMIM]OAc) was able to dissolve PBI more efficiently under low pressure and temperature, which was attributed to the strong ionic interaction of 1-ethyl-3-methylimidazolium acetate to break down the hydrogen bonds and π–π interaction in PBI [104]. The resulting membrane had a thick sponge-like structure with several macrovoids and exhibited decent performance for water flux (26.05 L·m^−2^·h^−1^) and solute rejection (>95%). Additionally, 1-ethyl-3-methylimidazolium acetate is miscible with water at any scale, making them prone to leaching after membrane casting for further recycling and reuse purposes.

In cellulosic membrane preparation, ionic liquids are among the very few solvent able to dissolve cellulose, mainly driven by its anionic groups. This greatly reduces the environmental issue as cellulose is the most abundant renewable resource, however, it is insoluble in most of traditional solvents. Ding et al. [105] were the first group of researchers who studied the fundamental differences in membrane formation between ionic liquid and traditional solvents (NMP and acetone) during the phase inversion of the CA membrane. The membrane formed using ionic liquid 1-butyl-3-methylimidazolium thiocyanate as a solvent, had a dense macrovoid-free structure full of nodules, indicating that the membrane formed via the slow process of nucleation growth and gelation. Then, the ionic liquids were recovered and reused. The resulting membrane formed using recovered solvent exhibited performance and morphologies similar to those prepared from the fresh solvent. Livazovic et al. [106] used 1-ethyl-3-methylimidazolium acetate to prepare multilayer membranes consisting of few polymers, and cellulose as the selective layer. The counterpart of silylated cellulose in tetrahydrofuran was produced as a benchmark. The cellulose silylation inhibits the formation of a hydrogen bond and creates hydrophobic cellulose soluble in tetrahydrofuran. The membrane prepared using ionic liquids had a water permeance of 13.8 L·m^−2^·h^−1^·bar^−1^ and a MWCO of 3000 g·mol^−1^, outperforming the silylated membrane in both water permeance (8.1 L·m^−2^·h^−1^·bar^−1^) and MWCO (5000 g·mol^−1^). The NaCl rejection of the latter was as low as 3%, which, however, was not being experimented with for a membrane prepared with ionic liquids. The cellulose membranes were also prepared without any polymeric supports and were confirmed to be insoluble in a series of traditional solvents. This proves the feasibility of using ionic liquids for cellulose dissolution, as well as the practicability of cellulosic membrane in various applications involving a solvent medium. On the other hand, ionic liquids such as 1-ethyl-3-methyimidazolium diethyl phosphate, 1,3-dimethylimidazolium dimethyl phosphate, and 1-butyl-3-methylimidazolium chloride were also employed by other groups of researcher as the solvent to prepare cellulosic membrane for dye, protein, and oil retentions [107,108,109]. The membrane performances showed that all ionic liquids can be good solvents for cellulose. Table 5 shows the summary of previous works from several authors using ionic liquids as solvents in membrane fabrication for desalination.

### 2.5. Other Potential Green Solvents

In addition, there are still numerous green solvents being used in membrane fabrication, such as glycerol triacetate (GTA) [110], triethylene glycol diacetate (TEGDA) [66], ATEC [65], ATBC [65], and methyl-5-(dimethylamino)-2-methyl-5-oxopentanoate (Rhodiasolv^®^ Polar-Clean) [111,112], Cyrene^TM^ [113], just that their application in desalination for the current state is scarcely found. However, as the whole scientific community is working harder towards achieving a sustainable green objective, it is expected that more exploration as well as studies for the great use of green solvents in membrane desalination will be available in the future.

## 3. Membrane Modification

Since the invention of membranes for desalination, these have undergone huge developments from time to time in terms of material science, synthesis methods, membrane modification, as well as process and system optimization [114]. Among the materials used to synthesize a desalination membrane, the PA-TFC membrane prepared via IP is by far the most popular due to its superior water permeability and salt rejection properties [115]. However, the permeability/rejection trade-off has affected the performance effectiveness. Efforts have been made to address such a bottleneck whereby membrane modification in general serves this purpose well. Alongside with the membrane community growth, tons of excellent, interesting and novel modification ideas and methods are gradually discovered to tackle the thorny challenge. Membrane modifications are flexible as different combinations and routes could potentially yield different outcomes to cater to the need. For instance, in the FO or RO process, it can be performed to enhance water flux and solute rejection, as well as to minimize fouling propensity, while in FO alone, it can solve the internal concentration polarization (ICP) phenomenon and reduce the reverse solute flux [116,117,118]. Although many modification approaches have been established, most of them require solvent. This review only focuses on sustainable approaches, which are mainly the solvent-free methods.

### 3.1. Click Chemistry for Membrane Modification

The click chemistry which was proposed in 2001 has become popular due to its robust and effective reactions [119]. It is an environmentally benign chemical reaction as it is mostly performed under non-stringent conditions such as room temperature and atmospheric pressure without consuming much external energy and producing inoffensive by-products which can be easily removed by non-chromatographic method (such as distillation and crystallization). Ideally, the process is insensitive to water and oxygen. There are wide scopes of reactants that can be used to carry out such reactions to produce a highly selective and highly yielded product (stereospecific) [119,120]. The reaction is known as the ‘‘spring loaded process’’ for a single trajectory as it has a thermodynamic driving force higher than 20 kcaL·mol^−1^ which makes the process rapid. The few most common click chemistries involving carbon–heteroatom bond forming include cycloaddition of unsaturated compound (1,3-dipolar cycloaddition), nucleophilic substitution reaction (ring opening reactions of strained heterocyclic electrophiles), carbonyl reaction of the ‘’non-aldol’’ compound, additions to carbon–carbon multiple bonds (epoxidation, aziridination, dihydroxylation, etc.). Among all, azide–alkyne 1,3-dipolar cycloaddition or Huisgen 1,3-dipolar cycloaddition is the most commonly employed reaction, which is the usually referred ‘‘click reaction’’ [121].

**Figure 6:**
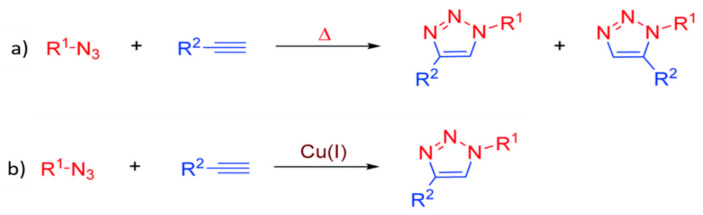
(**a**) Huisgen 1,3-dipolar cycloaddition; and (**b**) copper-catalyzed 1,3-dipolar cycloaddition [128].

**Figure 7:**
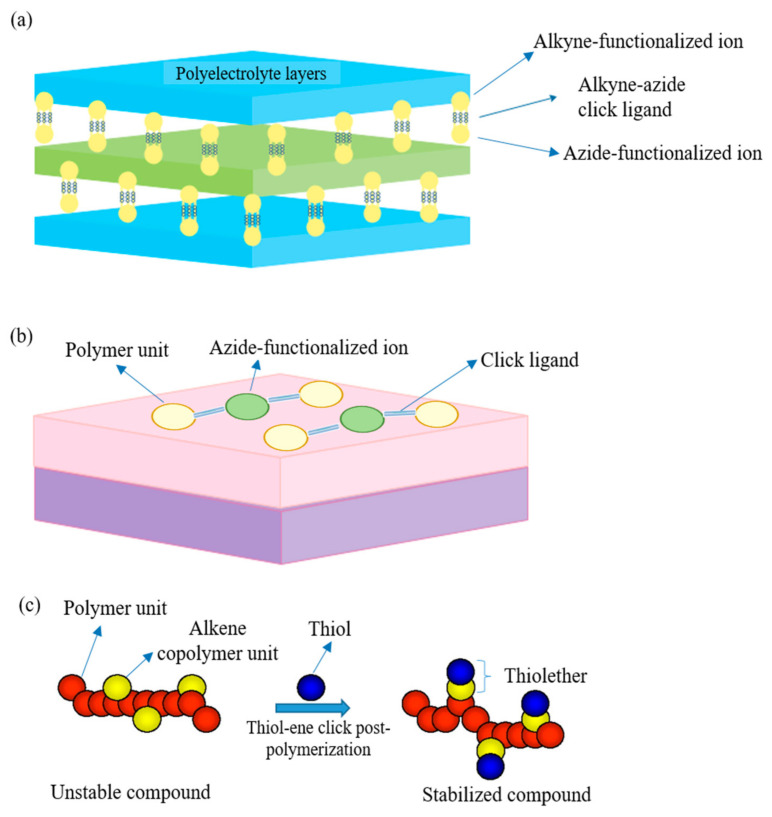
Click reaction in: (**a**) layer-by-layer (lbl) assembly technique; (**b**) nanofiller modification; and (**c**) post-polymerization modification.

The first breakthrough of the reaction was based on the introduction of copper (I) as a catalyst into the reaction in 2002 [122,123]. However, the copper-based reaction introduces cytotoxicity to the environment, especially to the biological system [124]. This potentially limits its application in certain practical situations, such as bioconjugation. Hence, copper (I)-free azide–alkyne 1,3-dipolar cycloaddition is introduced as a green alternative to overcome such a problem and has been widely adopted until today. However, the copper-free reaction comes with the compensation of being a rather slow, high temperature, and unselective process. Figure 6a,b show the cycloaddition reactions without and with copper (I) as the catalyst, respectively, whereby the equimolar mixture of 1,4- and 1,5-disubstituted 1,2,3-triazoles regioisomers are produced in the absence of copper. However, with a catalytic amount of copper (I), the reaction can be accelerated up to seven-fold with improved regioselectivity, forming 1,4- disubstituted isomer as the sole product. Lately, click chemistry is brought into membrane modification such as the nanofiller incorporation [125], post-polymerization modification [126], layer-by-layer (lbl) assembly [127], as an effective and eco-friendly approach to replace some current modification technologies. Figure 7a–c further illustrate the application of click reaction in different membrane modification techniques.

#### 3.1.1. Layer-by-Layer Assembly

Lbl assembly technique is a promising method to prepare membrane with an ultrathin selective layer which has good rejection towards divalent ions as well as strong thermal stability that has been successfully employed for NF operations throughout the years [129]. The lbl method involves the deposition of several polyelectrolytes with opposite charges onto porous substrate surface in alternate sequence. The first polyelectrolyte layer on the porous support layer is usually linked by electrostatic attraction or hydrophobic attraction. The lbl technique can be easily employed to produce composite polyelectrolyte multilayer membranes to obtain improved water flux and rejection. Despite the acceptable rejection for divalent ions such as Mg^2+^ and SO_4_^2−^, the retention of monovalent ions such as Na^+^ is remarkably low due to the relatively loose selective layer. As membrane fouling persists as one of the thorniest issues in RO operation, the strong surface charge of the selective layer is crucial to reduce membrane fouling. In order to potentially improve the membrane performances while minimizing fouling, Wang et al. [130] assembled polyethylene glycol (PEG) acrylate multilayers via the lbl technique on a commercial RO membrane and stabilized using click reaction. As the number of PEG bilayers increased, the fouling resistance of the membrane surface improved significantly. It is worth noting that the water flux did not suffer a severe decline owing to the addition of PEG bilayers, in which only a mere decline of 9–17% was observed. Additionally, the lbl assembly using click reaction was able to ensure the membrane thickness in the nanometer range for maximum effectiveness, as each PEG bilayer coated with click chemistry was only around 3.9 nm. Using such click chemistry to covalently bond the multiple films also grants the benefit of being ultra-stable, whereby the occurrence of disassembly is not likely to be observed under varying conditions (e.g., pH). From the seawater RO test, it was observed that despite undergoing 27 h operation, the resulting lbl membrane with 2-bilayer was able to retain a high water flux of around 110 L·m^−2^·h^−1^ and salt rejection of >94.5%.

Similarly, Cho et al. [130] fabricated a cross-linked lbl PSU membrane by spray coating with polyallylamine hydrochloride (PAH) and polyacrylic acid (PAA) polyelectrolytes. Azide–alkyne click reaction accounted for the polyelectrolytes cross-linking, which also removed the chemical or thermal post-coating treatment. The PAA was functionalized with alkyne while the PAH was functionalized with azide, and spray coating was performed for a duration of 20 min between each layer to allow the complete formation of 1,2,3-triazole linkage. The lbl assembly performed in such way that produced a polyelectrolyte layer with reduced coiling and thus smaller pores and improved rejection. The coating of the PAA polyanion layer resulted in increased water flux and decreased salt rejection, indicating the increase in membrane free volume. For the divalent CaCl_2_ salt, the rejection could be up to 80%; while for the monovalent NaCl salt, a rather unsatisfied rejection (50%) was observed. The study revealed that the structure of the resulting multilayer membrane was significantly influenced by its deposition method and the polyelectrolytes charge density. “Click” lbl deposition was proven as a feasible approach for NF desalination, however, for RO desalination, especially when dealing with monovalent salts, further development is required to hit a remarkable rejection height. A similar outcome was found in a study by Wang et al. [131], in which tubular ceramic multilayer membranes were fabricated via a clickable lbl assembly for dye desalination. PAA and poly(vinyl) alcohol were employed as the polyelectrolyte layers pre-cross-linked by glutaraldehyde (GA). The 96% Congo red rejection and 3% NaCl rejection indicated that the multilayer membrane was only feasible for NF but not RO application. The author concluded that a covalently clicked multilayer membrane has greater stability than a electrostatically bonded multilayer membrane.

#### 3.1.2. Modification with Nanomaterials

Generally, nanofiller modification serves the purpose of improving the surface hydrophilicity of a thin-film nanocomposite (TFN) membrane while reducing fouling. Meanwhile, it could also potentially affect the transport phenomena such as water transport and reverse solute diffusion. He et al. [128] prepared a novel aquaporin-based biomimetic PSU membrane using propargyl functionalized β-sheet peptide (FBP). To stabilize the aquaporin, the FBP was introduced to bind to it and form a stable peptide–protein complex in the aqueous medium, covalently conjugated with azide groups. The PSU polymers were first conjugated with a 1.05 azide group per repeating unit, followed by casting into a thin film support layer via NIPS. The peptide–protein complex was then “clicked” onto the support layer using a self-designed circulating system. The functional group analysis and surface morphology analysis indicated the successful incorporation of the peptide–protein complex. Compared to the previous strategies of incorporating aquaporin thin film onto membrane, the authors determined that by employing FBP and the click reaction, covalent bonding with stronger interaction and higher stability under high flow rate and pressure could be obtained. It was noticed that the salt rejection of the resulting membrane merely improved from 5 to 12.5%. Click reaction is feasible in conjugating a protein thin layer onto the polymeric support layer while maintaining its functionality. However, the suboptimal improvement of the salt rejection (12.5%) showed that there could be defects in the membrane, which might be from the low degree of click conjugation upon the pores directly. It can be accomplished by altering the casting condition or tightly binding several complexes in the membrane pores with optimized click reaction to obtain a support layer with a pore size similar to the protein complex.

**Figure 8:**
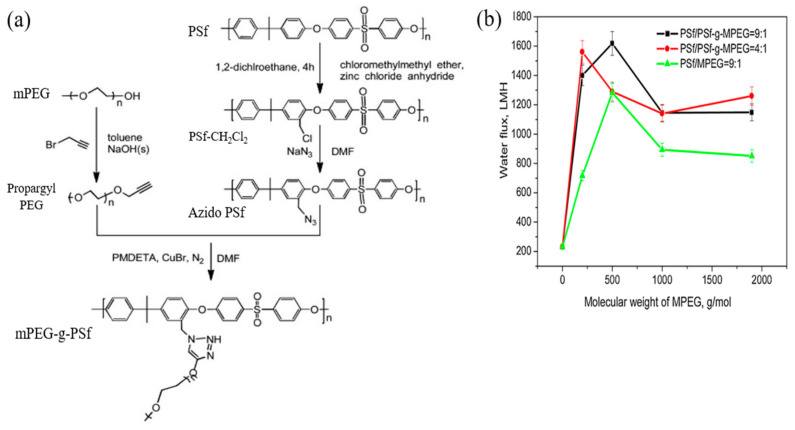
(**a**) Schematic representation of the preparation of alkynyl- methoxypolyethylene glycol (mPEG), chlorinated PSU, azido-PSU and the click reaction between alkynyl-mPEG and azido-PSU to obtain mPEG-g-PSU; and (**b**) the water flux test for the substrate membrane [135].

For FO application, click modification often offers more advantages such as reducing structural parameters and ICP in addition to granting water permeability and antifouling performance [132]. The structural parameter is the quantitative measurement used to indicate the level of ICP, while ICP is a highly undesirable phenomenon in FO which can lead to very poor FO efficiency by limiting the water flux up to 99.9% [133,134]. Zhou et al. [135] prepared a TFC FO membrane via click reaction for structural parameter reduction. Modification was done on the PSU substrate by blending or click-grafting it with different molecular weights and ratios of methoxypolyethylene glycol (mPEG). Precedent procedures were carried out on mPEG and PSU prior to the click-grafting of mPEG onto PSU to form a PSU-g-mPEG substrate as illustrated in Figure 8a. The prepared substrate was then coated with a PA layer via IP forming the TFC membrane. Compared to the mPEG-PSU membrane prepared using the blending technique, the click-grafted membrane offers more stable and higher water flux which can be seen from the flux test result in Figure 8b, due to the fact that mPEG could more easily concentrate on the membrane skin and inner pore surfaces, and harder to leach out during the filtration process. Compared to the pristine TFC membrane, the click-modified TFC membrane exhibited a sharp drop in structural parameters and a significant improvement in pure water flux due to the hydrophilic click-grafting of mPEG. Meanwhile, the salt rejection remained the same for all membranes, modified or unmodified, the value of which was not mentioned in the study.

Soroush et al. [136] incorporated Ag–graphene oxide (GO) onto a commercial FO TFC membrane to impart hydrophilicity and anti-biofouling properties to the membrane. Cysteamine solution was used to functionalize the surface of the TFC membrane to provide a membrane surface with thiol functionality for click reaction between Ag–GO and amine groups of cysteamine. The functionalization was successful when the unreacted acyl halide groups from the PA layer reacted with the amine groups of cysteamine via click reaction forming strong amide bonds. The approach is more effective than the conventional modification approaches as just a lower material cost and easier preparation method are required. Minor flux reduction was observed compared to the pristine TFC due to the formation of an extra Ag–GO layer on the membrane surface which hinders the water transport mechanism. Nonetheless, remarkable anti-biofouling improvement was observed, whereby the modified membrane had a bacterial inactivation of >95% compared to ~0% of the pristine TFC membrane.

**Figure 9:**
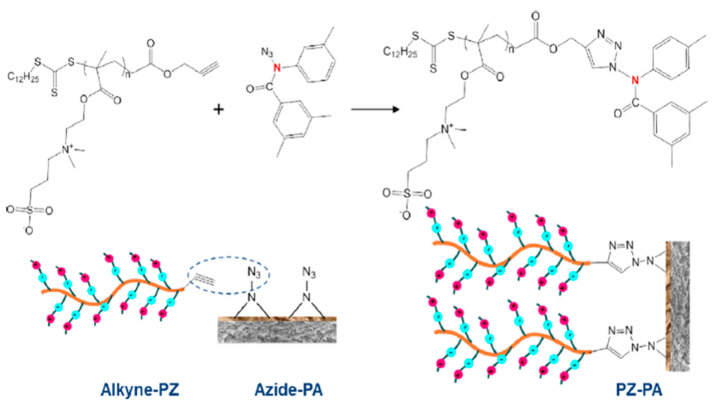
Click chemistry of azide–PA and alkyne–polyzwitterions (PZs) with their respective chemical structure shown on top.

Meanwhile, Yu et al. [137] grafted polyzwitterions (PZs) onto the TFC membrane surface using click chemistry to improve its antifouling performance for FO desalination. Click chemistry introduces a much milder approach in contrast to the harsh conditions usually needed by many traditional grafting methods, in which SN2 nucleophilic substitution on nitrogen of the PA chain could occur under mild conditions. Briefly, alkyne–PZs was first prepared via reversible addition–fragmentation chain-transfer radical polymerization. Then, the PA layer was brominated followed by the SN2 nucleophilic substitution of the Br group with an azide functional group. Finally, the alkyne–PZs were grafted onto the azide–PA via cycloaddition click reaction (Figure 9). The water flux of the PZs-TFC membrane was fairly affected by the click grafting, and instead, the antifouling properties of the resulting membrane improved tremendously due to the hydration layer which acted as a barrier on the membrane surface, the strong hydrophilic repulsion caused by the charge-induced hydration force, and the steric hindrance by the brush-like bulky PZ chains. The flux recovery of the PZs-TFC membrane was nearly 100% while that of the control membrane was only 77%.

#### 3.1.3. Post-Polymerization Modification

Often, post-polymerization modification is required to stabilize the polymer product as it could be unstable and become reversible. For instance, Yang et al. [129] used thiolene click reaction to carry out a post-polymerization modification for preparing a zwitterionic PSU-TFC membrane with superior hydrophilicity and antifouling behavior for the RO desalination. The purpose of the post-polymerization click modification was mainly to ensure the stability of the zwitterion copolymer formed. At first, the tertiary amines and ring-opened sultone were successfully introduced to a zwitterion forming copolymer. The thiolene click reaction successfully isolated the tertiary amine-modified zwitterion copolymer. Most importantly, the resulting membrane prepared from the click reaction had no unsaturated bonds from the allyl group or the corresponding isomer which makes it very stable at all conditions. Additionally, the resulting membrane exhibited a decent performance in terms of water flux (2.5 L·m^−2^·h^−1^) and NaCl rejection above 98% even under high chlorine exposure. The hydration layer induced by the stabilized zwitterion copolymer successfully imparted antifouling capability to the membrane, granting a high flux recovery up to 94%. Table 6 summarizes the recent works involved in click chemistry for desalination membrane modification.

### 3.2. Chemical Vapor Deposition for Membrane Modification

**Figure 10:**
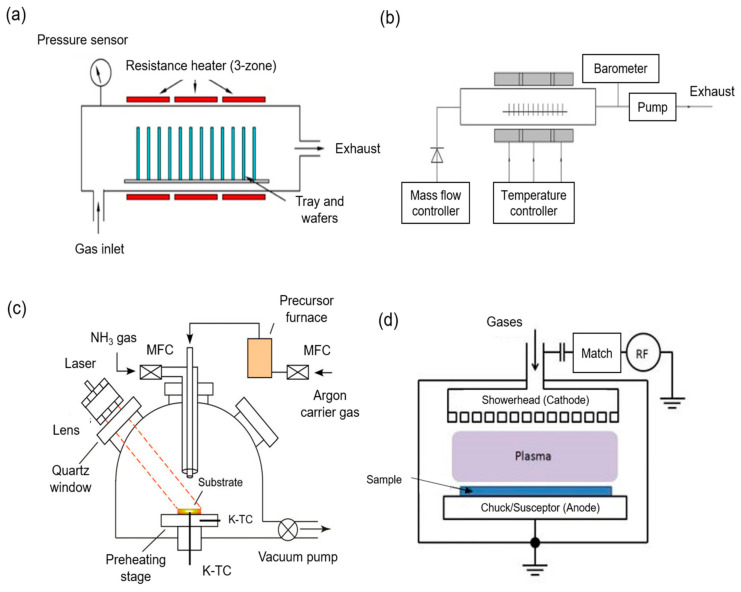
Configuration setup for (**a**) atmospheric chemical vapor deposition (CVD); (**b**) low pressure CVD; (**c**) laser CVD; and (**d**) plasma-enhanced CVD (PECVD) [143,144].

Chemical vapor deposition (CVD) is a chemical decomposition process whereby a vapor precursor is converted into solid material and coated onto a substrate surface upon heating [138]. There are many kinds of CVD processes, such as atmospheric pressure CVD, low pressure CVD, laser CVD, and plasma-enhanced CVD (PECVD) [139], as depicted in Figure 10. The energy required to initiate the polymerization process for CVD originates from various sources, such as plasma species for PECVD and laser for laser CVD [140,141]. The solid product is commonly in the form of thin film, single crystal or powder. Compared to many traditional methods, CVD is much more versatile as a wide range of chemical, physical, and even the tribological properties of materials can be produced by manipulating experimental conditions such as reaction gas mixture composition, total gas pressure, substrate temperature and substrate material. [138]. The other advantages of CVD include large area growth coverage, conformal growth, high reproducibility, and a high purity product [142]. Most importantly, the whole process does not require solvent, making it an environmentally friendly process.

To date, CVD has been widely used in thin film-related applications, as it can produce coating with uniform thickness and low porosity even on a substrate with a complicated shape [138]. Hence, its feasibility in membrane application has been investigated by some researchers. Among all the CVD processes, a novel method, namely initiated CVD (iCVD) has gained attraction, whereby the deposition of materials is completed via free-radical polymerization induced by gas phase radicals at low operating pressure and temperature without employing any solvent. Ozaydin-Ince et al. [145] used iCVD to coat the amphiphilic copolymer films of hydrophobic perfluorodecylacrylate (PFA) hydrophilic hydroxyethylmethacrylate on commercial RO membranes to cope with membrane biofouling, which is a prickly issue for TFC membrane. Using the iCVD technique, the membrane was modified without the active layer being damaged. An optimum composition of the copolymer films was experimented with and obtained to achieve a high membrane performance and reduce cells’ attachment, with a coating thickness of 20 nm as the most optimum flux performance. Compared to the control of commercial membranes, the modified membranes with 20 nm coating had a 10% water flux lower owing to the additional layer, and even lower to 50% with 50 nm coating. The salt rejection was maintained at 98% for both coated and control membranes, which, however, was not affected by the coating chemistry. The rather insignificant flux drop was then compensated by a great improvement in the membrane antibiofouling performance, whereby the cell attachment on the surface of the modified membrane was greatly reduced after 1 h of experiment.

A similar outcome was observed by Matin et al. [146], where iCVD was employed to modify the surface of commercial RO membranes. 2-hydroxyethyl methacrylate-co-perfluorodecyl acrylate (HEMA-co-PFDA) copolymer films was introduced to the membrane surface via iCVD for sodium alginate fouling reduction. The coated membrane exhibited a much lower water contact angle, indicating that hydrophilicity was imparted from the copolymer films. Moreover, the flux reduction in the modified membrane is much lower than the control membrane, while the salt rejection was nearly unaltered. Even though fouling appears to be an inevitable phenomenon in membrane process, however, it can be potentially delayed by iCVD surface modification. Kazemi et al. [147] prepared large area graphene membrane for desalination application. The graphene membrane was fabricated via CVD to control the porosity. CVD-grown graphene, however, possesses some inevitable characteristics such as intrinsic defect, cracked hole, and wrinkles which could impact the membrane performance adversely, hence hole arrays in silicon were employed as the secondary support. The resulting membrane exhibited decent water permeation and varied salt rejection (58−100%). The highly varied salt rejections were attributed to the different array diameters and numbers. In short, the lower rejections were advantageous in terms of selectivity when dealing with solution containing multiple ions or bigger size ions granting high permeation, while the high rejections were ideal for water desalination.

On the other hand, Lai et al. [148] employed PECVD for the incorporation of poly (hexafluorobutyl acrylate)-modified hydrous manganese oxide (PHFBA-modified HMO) nanomaterials in TFN membrane for NF desalination. The polymerization reaction involves reactions between plasma species, surface and plasma species, and surface and surface species [149]. However, in this study, PECVD was not only served as a green approach for membrane modification, instead it was also effective in enhancing the dispersibility of HMO in solvent reducing agglomeration. The even dispersion of the modified HMO over the membrane surface led to remarkable water permeability enhancement, with 0.5 *w*/*v* % HMO as the optimum amount for the highest water permeability which was 66.6% higher than the control TFC. The modified HMO incorporation also significantly improved the membrane surface negativity and PA cross-linking degree, resulting in excellent salt rejections (Na_2_SO_4_: 98.6%; MgSO_4_: 97.6%). In addition, the resulting TFN membrane also exhibited remarkable fouling resistance improvement against organic and inorganic foulants.

### 3.3. Plasma Treatment for Membrane Modification

To improve the performance of membrane desalination, the incorporation of nanofillers plays a vital role as it undeniably grants certain desirable traits to the membrane such as permeability and selectivity. However, before it finally reaches the form where it can be highly desirable, there are always some unwanted paradox to deal with. For instance, most nanofillers have a natural tendency to agglomerate forming large clusters, further inducing stress in the membrane composite and degrading its properties [150,151]. Additionally, due to the possibly different physicochemical properties of nanofillers and polymer, it often results in poor compatibility between them. Therefore, nanofillers or membrane surface modification becomes an indispensable step before dispersing into the polymer matrix in order to fully minimize the agglomeration and improve the matrix compatibility.

Plasma treatment emerges as an effortless yet effective method to cope with this problem. Notably, the use of solvent is not needed throughout the process, making it a green method that fulfils the objective of this study. In brief, the plasma treatment creates a reactive environment which consists of radicals, electrons, ions, and excited molecules [152]. As soon as the interaction with a solid surface occurs, the process is complete right after the transfer of energy from the plasma particles through elastic and inelastic collisions which significantly induce physical and/or chemical changes in that surface. Plasma treatment is a versatile technique that can be applied for a wide range of materials such as polymer and inorganic nanomaterials. Since the performance of membrane separation such as water flux and rejection mainly depend on the size exclusion and membrane surface characteristics, the range of applications can be potentially improved via plasma treatment [153].

In a pioneering study by Wu et al. [154], the chlorine resistance and the water permeability of the prepared RO membrane was significantly improved by plasma treatment with oxygen and argon plasma at high discharge power. The oxygen plasma successfully introduced the carboxyl group to the membrane surface, increasing permeability while cross-linking induced by argon plasma at the nitrogen site of PA improved the membrane chlorine resistance. As commercial membranes often face difficulties in rejecting multivalent cations and specific ionic species owing to surface negativity, Déon et al. [155] grafted allylamine on commercial PA membrane using plasma treatment modification. The plasma-treated membrane possessed high selectivity for monovalent and divalent ions regardless of their charges, exhibiting excellent solutes rejection. The astonishing rejection performances and selectivity of the resulting membranes were caused by the partial coverage of poly(allylamine), whereby 5 and 10 min of polymerization yielded membranes with a slightly negative or slightly positive overall apparent zeta potential. Meanwhile, the water permeability was marginally affected by the plasma treatment, whereby the modified membrane experienced a 20% lower water permeability compared to the pristine membrane. This phenomenon was due to a decrease in membrane mean pore size by around 60% after plasma-assisted grafting.

On the other hand, Ohland et al. [156] functionalized hydroxyapatite (Hap) particles with argon plasma treatment to insert hydroxyl groups onto the surface and further incorporated into CA membrane. A huge leap of water permeability (0.39−0.51 L·h^−1^·m^−2^·bar^−1^) was achieved while maintaining a high level of MgSO_4_ and NaCl rejections in RO desalination system. When tested in the FO system, the water flux was also enhanced by 66% from 4.5 to 7.5 L·h^−1^·m^−2^ with a low reverse salt flux consistently maintained. In short, it is safe to conclude that the plasma treatment and hydroxyl group insertion successfully improved the membrane hydrophilicity without adversely disrupting the membrane morphology. Woo et al. [157] fabricated an omniphobic PVDF membrane by electrospinning and plasma treatment with the CF_4_ plasma for air gap MD brine treatment. From the morphological studies, it was observed that the formation of new CF_3_ and CF_2_–CF_2_ bonds from the plasma treatment had successfully lowered the surface energy of the membrane granting omniphobic properties and wetting resistance. Regarding the performance tests, the resulting membrane exhibited stable normalized flux and great solute rejection even after adding 0.7 mM of sodium dodecyl sulphate (SDS) to the brine feed as surfactant. The commercial PVDF membrane on the contrary, suffered from surface wetting after the addition of 0.3 mM SDS.

## 4. Future Challenges and Conclusions

Although fresh water scarcity has become a gradually developing chronic issue in the world, the majority the people still have less concern and less awareness of this issue, preventing the development pace from catching up with the extent of severity. Membrane desalination as an emerging technology has great potential to overcome the water scarcity issue with high efficiency. However, the traditional membrane preparation methods usually involve the use of toxic solvents which carry the danger of eco-toxicity, hence replacing the traditional solvents with some non-toxic or less toxic solvents is highly desirable. It is not merely a suggestion but also a need to find greener alternatives to traditional solvents, as the European Union has started to restrict the use of toxic organic solvents in 2020. For instance, NMP usage cannot exceed a concentration of 0.3%, excepting that both manufacturer and consumer parties are willing to undergo appropriate risk management protocols [61]. Obviously, other traditional solvents have a similar fate awaiting them. Soon, it is expected that the solvent restrictions will be imposed on every country in the world. Many of the industries involving solvents will be harshly affected. Nonetheless, switching to green solvents that complies with the regulation have some challenges to be overcome, mainly impaired performance and price competitiveness. Specifically in the membrane industry, it is difficult to find solvents which produce membranes with on par performance with those prepared using well-established solvents like NMP. Some green solvents might give a similar performance to the traditional ones, yet the production routes are still costly, such as ionic liquids. In fact, there are reasons why traditional solvents have been widely employed until today despite their well-known toxicity, because they simply offer more advantages compared to the greener alternatives: particularly, unproducible in the large scale, limited dissolution, limited production area, high viscosity, and high cost. Fortunately, results from some research gave indication that it is completely possible to achieve these goals simultaneously, not yet now but very soon. In the future, it is expected that more concerted efforts will be done by the research and scientific communities to overcome these obstacles, while the solvent selection trend will be either inclined towards extracting bio-solvent from the nature such as plants or animals which is completely non-toxic, or the solvent-free approach as the ultimate green goal.

Meanwhile, membrane modification which produces harmful by-products can be minimized by using solvent-free techniques such as CVD or plasma treatment, or utilizing click chemistry reaction which is mostly performed in non-stringent conditions without consuming much external energy and producing an inoffensive bi-product. A challenge faced is the inability of CVD to produce large area defect-free materials such as large area graphene sheets, which are in high demand but not commercially available yet in the market. Then, it creates new prospects and insights in developing the growth of large-area high-quality defect-free materials with desired traits, such as excellent dispersion, well-defined sizes and fine control on layer thickness in the future. Meanwhile, in click-chemistry, despite being all good in terms of the green approach with great efficiency, optimization is still required to overcome certain challenges such as the alkyne homocoupling, whereby alkyne reacts with other alkyne groups instead of the azide group. Some click reactions require a metal catalyst such as copper to avoid producing by-products, and long-term exposure to the metal could lead to physiological side effects such as neurologic and renal diseases. Hence, in the future, it is expected to discover more reaction routes such as the substitution of the azide group with other less hazardous species.

In a nutshell, although membrane processes are recognized as green processes, traditional membrane fabrication methods are rather hazardous due to the toxic solvents employed throughout the fabrication steps such as phase inversion, membrane modification, and post-treatment. With the lead of green chemistry principles which advocate the use of materials with low environmental impacts, it is definitely possible to prepare the desalination membrane of desired morphologies and performances in a much more sustainable approach. Particularly, during phase inversion, non-toxic solvents categorized into organic synthetic solvents, bio-sourced solvents, and ionic liquids are used to replace the traditional ones with high toxicity. Nevertheless, it is important to know that while environmental advantages alone are not sufficient for the widespread adoption of green solvents, factors like performances and costs also make their counts. While for membrane modification processes, the employment of solvent-free techniques such as click reaction, CVD or plasma treatment are greatly encouraged. The ease of these techniques creates new insight for the maximum production and efficacy of membranes with minimum environmental impacts. With more works being published, the era of membrane technologies is shifting towards sustainability. In the future, the consideration of sustainability will definitely be one of the criteria for judging membrane performances.

## Figures and Tables

**Table 1:** Hazard classification of the solvents commonly used for substrate fabrication [24].

Solvent	Boiling Point (°C)	Fabrication Method	Hazard and Toxicological Statements
Dimethylformamide (DMF)	153	NIPS	Flammable liquid and vapor. Harmful in contact with skin or if inhaled. Causes severe eye irritation. Damages the unborn child. Causes mutations in germ cells and mammalian somatic cells.
Dimethylacetamide (DMA)	166	NIPS	Harmful in contact with skin or if inhaled. Causes serious eye irritation. May damage the unborn child. Causes congenital malformation to the fetus. Produces human reproductive toxicant and causes reproductive disorders.
*N*-methyl-2-pyrrolidone (NMP)	202	NIPS/TIPS	Causes skin irritation. Causes serious eye irritation. Cause respiratory irritation. May damage the unborn child. May damage the fetus.
1,4-Dioxane	101	NIPS	Highly flammable liquid and vapor. Causes serious eye irritation. May cause respiratory irritation. Suspected of causing cancer. Forms explosive peroxides. May cause skin dryness or cracking.
Dibutyl-phtalate (DBP)	384	TIPS	May damage the unborn child. Extremely toxic to aquatic life.
Dioctyl-phtalate (DOP)	384	TIPS	Cause infertility. May damage the unborn child. Causes congenital malformation in the fetus. Produces human reproductive toxicant and causes reproductive disorders.
Acetone	56	NIPS	Highly flammable liquid and vapor. Causes serious eye irritation. May cause drowsiness or dizziness.
Chloroform	61.2	NIPS	Harmful if swallowed or if inhaled. Causes skin irritation effect. Causes serious eye irritation. May cause drowsiness or dizziness. Suspected of causing cancer. Suspected of damaging the unborn child. Causes germ cell mutation. May cause damage to organs through prolonged or repeated exposure.
Tetrahydrofuran (THF)	66	NIPS	Highly flammable liquid and vapor. Causes serious eye irritation. May cause respiratory irritation. May lead to cancer.
Toluene	111	NIPS	Highly flammable liquid and vapor. May be fatal if swallowed and enters airways. Causes skin irritation. May cause drowsiness or dizziness. May damage fetus. May cause damage to organs through prolonged or repeated exposure. Brings reproductive toxicity to male and female animals.

**Table 2:** HSP values of the polymers and the solvents, and the radius of interaction of the Hansen solubility sphere (R_0_).

Polymers	δ_d_ (MPa^1/2^)	δ_p_ (MPa^1/2^)	δ_h_ (MPa^1/2^)	R_0_ (MPa^1/2^)	Ref.
Cellulose acetate (CA)	18.6	12.7	11	8.8	[62]
Polyvinylidene fluoride (PVDF)	17.2	12.5	9.2	5	[63]
Polysulfone (PSU)	19.7	8.3	8.3	8	[62]
Polyvinyl chloride (PVC)	17.6	7.8	3.4	8.2	[62]
Polyacrylonitrile (PAN)	21.7	14.1	9.1	10.9	[62]
Chitosan	21.9	32.5	24.6	NA	[60]
Solvents					
Acetyltributylcitrate (ATBC)	16.02	2.56	8.55	−	[64]
Acetyltriethylcitrate (ATEC)	16.6	3.5	8.6	−	[65]
Triethyl citrate (TEC)	16.5	4.9	12	−	[65]
Triethylene glycol diacetate (TEGDA)	16.45	2.14	9.78	−	[66]
Cyrene	18.8	10.6	6.9	−	[67]
Dimethyl isosorbide (DMI)	17.6	7.1	7.5	−	[53]
TamiSolve NxG	17.8	8.2	5.9	−	[68]
Ethyl lactate	16	7.6	12.5	−	[62]
Methyl lactate	15.8	6.5	10.2	−	[69]
PolarClean	15.8	10.7	9.2	−	[63]
Dimethylsulfoxide (DMSO)	18.4	16.4	10.2	−	[70]
Triethylphosphate (TEP)	16.8	11.5	9.2	−	[71]
γ-butyrolactone	19	16.6	7.4	−	[64]
Propylene carbonate (PC)	20	18	4.1	−	[60]

**Table 3:** Characteristics of synthetic organic solvents and their summarized studies in membrane desalination.

Solvents	Chemical Structure		Boiling Point (°C)	Green Credentials		
DMSO ((CH_3_)_2_SO)	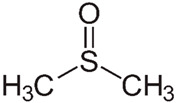		189	High boiling point Low vapor pressure Water miscible		
Membrane (Technology)	Fabrication Technique	Solvents	Feed Conc. (g·L^−1^)	Water flux (L·m^−2^·h^−1^)	Salt rejection (%)	Ref.
CTA/CNCs (PV)	NIPS	DMSO Dioxane NMP DMF	30.0 and 90.0	11.67 11.99 11.68 8.08	All > 99.0 (30.0 g·L^−1^ NaCl) DMSO > 99.0 (90.0 g·L^−1^ NaCl)	[74]
CTA/Al_2_O_3_ (PV)	NIPS	DMSO	30.0	6.70	99.8 (NaCl)	[75]
ANF-PA (NF)	NIPS	DMSO	1.0	57.60	100.0 (Na_2_SO_4_) 99.4 (MgSO_4_) 92.7 (MgCl_2_) 80.3 (NaCl)	[76]
PAN/Zeolite 13× (PV)	NIPS	DMSO DMF DMA	~50 wt.%	27.70 61.14 3.45	Highest (DMSO)	[79]
Solvents	Chemical Structure		Boiling Point (°C)	Green Credentials		
DMC (C_3_H_6_O_3_)	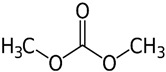		90	Low vapor pressure		
Membrane (Technology)	Fabrication technique	Solvents	Feed conc. (g·L^−1^)	Water flux (L·m^−2^·h^−1^)	Salt rejection (%)	Ref.
PSU-PA (RO)	NIPS	DMC	2.0	64.2	99.03 (NaCl)	[80]
Solvents	Chemical Structure		Boiling Point (°C)	Green Credentials		
TamiSolve NxG (Proprietary)	(Proprietary)		108 (Closed cup)	High boiling point Low vapor pressure Water miscible		
Membrane (Technology)	Fabrication technique	Solvents	Feed conc. (g·L^−1^)	Water flux (L·m^−2^·h^−1^)	Salt rejection (%)	Ref.
PVDF-HFP (DCMD)	NIPS	TamiSolve NxG	21.3	25	99.5	[68]

**Table 4:** The characteristics of synthetic organic solvents and their summarized studies in membrane desalination.

Solvents	Chemical Structure		Boiling Point (°C)	Green Credentials		
Methyl lactate (C_4_H_8_O_3_)	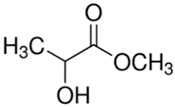		144	Biodegradable Water miscible		
Membrane (Technology)	Fabrication technique	Solvents	Feed conc. (g·L^−1^)	Water flux (L·m^−2^·h^−1^)	Salt rejection (%)	Ref.
CA (NF)	NIPS	Methyl lactate/ 2-Methyl THF (Co-solvent)	0.6	134.4	80.2 (MgSO_4_)	[69]
Solvents	Chemical Structure		Boiling Point (°C)	Green Credentials		
γ-Butyrolactone (C_4_H_6_O_2_)	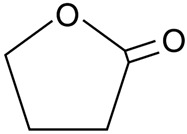		204	High boiling point Low vapor pressure Biomass derivation Biodegradable		
Membrane (Technology)	Fabrication technique	Solvents	Feed conc. (g·L^−1^)	Water flux (L·m^−2^·h^−1^)	Salt rejection (%)	Ref.
PEEK-WC (HFMC)	NIPS	γ-Butyrolactone	0.01–0.1	−	99.0 (Cr salt)	[85]
PVDF (DCMD)	TIPS	γ-Butyrolactone	3.5 wt.%	51.5	99.99 (NaCl)	[84]
Solvents	Chemical Structure		Boiling Point (°C)	Green Credentials		
ATBC (C_20_H_34_O_8_)	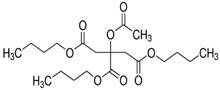		327	Biodegradable High boiling point Low vapor pressure		
Membrane (Technology)	Fabrication technique	Solvents	Feed conc. (g·L^−1^)	Water flux (L·m^−2^·h^−1^)	Salt rejection (%)	Ref.
PVDF (DCMD)	TIPS	ATBC	3.5 wt.%	~21	99.9 (NaCl)	[89]
ECTFE (VMD)	TIPS	ATBC	3.5 wt.%	22.3	99.9 (NaCl)	[90]
Solvents	Chemical Structure		Boiling Point (°C)	Green Credentials		
Soybean Oil (C_57_H_98_O_12_)	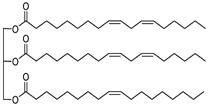		300	Biomass derivation Biodegradable High boiling point		
Membrane (Technology)	Fabrication technique	Solvents	Feed conc. (g·L^−1^)	Water flux (L·m^−2^·h^−1^)	Salt rejection (%)	Ref.
IPP (VMD)	TIPS	Soybean oil	29.22	24.81	>99.9 (NaCl)	[95]
PP (VMD)	TIPS	Soybean oil/carnauba wax	10	41.2	99.95 (NaCl)	[96]

**Table 5:** Characteristics of ionic liquids and their summarized studies in membrane desalination.

Solvents	Chemical Structure		Boiling Point (°C)	Green Credentials		
[Emim]OAc (C_8_H_14_N_2_O_2_)	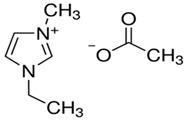		164	Complete reduction under mild conditions Negligible vapor pressure High boiling point		
Membrane (Technology)	Fabrication technique	Solvents	Feed conc. (g·L^−1^)	Water flux (L·m^−2^·h^−1^)	Salt rejection (%)	Ref.
PBI (NF)	NIPS	[Emim]OAc	0.2	26.05	>95.0 (MgSO_4_)	[103]
Silylated cellulose Cellulosic	Coating on multilayers NIPS	THF/ Acid treatment [Emim]OAc	2 2	40.5 69.0	3.0 (NaCl) N/A	[106]

**Table 6:** Summary of click chemistry for membrane modification.

Application	Membrane	Click Approach	Feed Solution	Water Flux (L·m^−2^·h^−1^)	Salt Rejection (%)	Flux Recovery (%)	Ref.
NF	Lbl PAA/PVA ceramic membrane	Click reaction to link PAA and PVA layers	1 g·L^−1^ NaCl	~25.0	3.0	N/A	[131]
NF	Lbl PAA/PAH PSU membrane	Click reaction to link PAA and PAH layers	32 g·L^−1^ NaCl 2 g·L^−1^ CaCl_2_	~300.0	~50 (NaCl) ~80 (CaCl_2_)	N/A	[130]
RO	Lbl PEG TFC membrane	Click reaction to stabilize PEG multilayers	30.83 g·L^−1^ NaCl	~110.0	>94.5	N/A	[127]
RO	SBAES zwitterionic TFC membrane	Clicked post-polymerization for PAES copolymer	2.0 g·L^−1^ NaCl	27.2	~98	94	[129]
RO	Aquaporin-FBP TFC membrane	Click reaction to link FBP and PSU substrate	1.0 g·L^−1^ NaCl	~4.53	12.5	N/A	[128]
FO	mPEG TFC membrane	Click reaction to graft mPEG onto PSU substrate	NaCl	~2.5	High	N/A	[135]
FO	Ag–GO TFC membrane	Click reaction to graft Ag–GO onto PA layer	50 mM NaCl	~5.5	~95	N/A	[136]
FO	PZs TFC membrane	Click reaction to graft PZs onto PA layer	20 mM NaCl	~5.4	High	~100	[137]

## Data Availability

Not applicable.

## Abbreviations

MSF	Multi-stage flash
RO	Reverse osmosis
MED	Multi-effect distillation
NF	Nanofiltration
FS	Feed solution
MD	Membrane distillation
FO	Forward osmosis
DS	Draw solution
UF	Ultration filtration
TFC	Thin film composite
IP	Interfacial polymerization
NMP	*N*-methyl-2-pyrrolidone
DMA	*N*,*N*-dimethylacetamide
DMF	*N*,*N*-dimethylformamide
THF	Tetrahydrofuran
TIPS	Temperature-induced phase separation
NIPS	Non-solvent induced phase separation
EIPS	Evaporation-induced phase inversion
VIPS	Vapor-induced phase inversion
DMSO	Dimethyl sulfoxide
ATBC	Acetyltributylcitrate
ATEC	Acetyltriethylcitrate
HSP	Hansen solubility parameter
VOC	Volatile organic compounds
CNCs	Cellulose nanocrystals
CTA	Cellulose triacetate
PA	Polyamide
ANF	Kevlar aramid nanofiber
DMC	Dimethyl carbonate
PVDF-HFP	Poly(vinylidene fluoride-hexafluoropropylene)
DCMD	Direct contact membrane distillation
MWCO	Molecular weight cut off
DOP	Dioctyl phthalate
PEEK-WC	Polyether ether ketone
HFMC	Hollow fiber membrane contractor
ECTFE	Poly(ethylene chlorotrifluoroethylene)
DEP	Diethyl phthalate
PBI	Polybenzimidazole
([EMIM]OAc)	1-ethyl-3-methylimidazolium acetate
GTA	Glycerol triacetate
ICP	Internal concentration polarization
Lbl	Layer-by-layer
PEG	Polyethylene glycol
PAH	Polyallylamine hydrochloride
PAA	Polyacrylic acid
GA	Glutaraldehyde
TFN	Thin film nanocomposite
FBP	Functionalized β-sheet peptide
mPEG	Methoxypolyethylene glycol
PZs	Polyzwitterions
GO	Graphene oxide
CVD	Chemical vapor deposition
PECVD	Plasma-enhanced chemical vapor deposition
iCVD	Initiated chemical vapor deposition
PFA	Perfluorodecylacrylate
HEMA-co-PFDA	2-hydroxyethyl methacrylate-co-perfluorodecyl acrylate
PHFBA-modified HMO	Poly (hexafluorobutyl acrylate)-modified hydrous manganese oxide
Hap	Hydroxyapatite
